# The Effects of Single and Combined Stressors on Daphnids—Enzyme Markers of Physiology and Metabolomics Validate the Impact of Pollution

**DOI:** 10.3390/toxics10100604

**Published:** 2022-10-12

**Authors:** Anna Michalaki, Allan Robert McGivern, Gernot Poschet, Michael Büttner, Rolf Altenburger, Konstantinos Grintzalis

**Affiliations:** 1 School of Biotechnology, Dublin City University, D09 Y5NO Dublin, Ireland; 2 Centre for Organismal Studies (COS), Heidelberg University, 69120 Heidelberg, Germany; 3 Department of Bioanalytical Ecotoxicology, Helmholtz-Centre for Environmental Research—UFZ, 04318 Leipzig, Germany

**Keywords:** *Daphnia magna*, mixture toxicology, combined stressors, mortality, biochemical markers, metabolomics

## Abstract

The continuous global increase in population and consumption of resources due to human activities has had a significant impact on the environment. Therefore, assessment of environmental exposure to toxic chemicals as well as their impact on biological systems is of significant importance. Freshwater systems are currently under threat and monitored; however, current methods for pollution assessment can neither provide mechanistic insight nor predict adverse effects from complex pollution. Using daphnids as a bioindicator, we assessed the impact in acute exposures of eight individual chemicals and specifically two metals, four pharmaceuticals, a pesticide and a stimulant, and their composite mixture combining phenotypic, biochemical and metabolic markers of physiology. Toxicity levels were in the same order of magnitude and significantly enhanced in the composite mixture. Results from individual chemicals showed distinct biochemical responses for key enzyme activities such as phosphatases, lipase, peptidase, β-galactosidase and glutathione-S-transferase. Following this, a more realistic mixture scenario was assessed with the aforementioned enzyme markers and a metabolomic approach. A clear dose-dependent effect for the composite mixture was validated with enzyme markers of physiology, and the metabolomic analysis verified the effects observed, thus providing a sensitive metrics in metabolite perturbations. Our study highlights that sensitive enzyme markers can be used in advance on the design of metabolic and holistic assays to guide the selection of chemicals and the trajectory of the study, while providing mechanistic insight. In the future this could prove to become a useful tool for understanding and predicting freshwater pollution.

## 1. Introduction

Humans have a significant impact on the physical environment in many ways such as overpopulation, pollution, fossil fuels and deforestation, which are responsible for the observed climate change, increased pollution, and decrease in air, water and soil quality. Therefore, assessment of environmental exposure to toxic chemicals as well as their impact on biological systems is of significant importance. Until recently, most approaches in water monitoring were based on the detection of individual chemicals, the physicochemical and microbiological parameters in a typical water analysis [1] and the determination of abundance and diversity of fauna and flora, which were subsequently compared against water quality standards [2]. However, these measurements have several weaknesses such as their cost for analytical quantification, the limited detection of a small number of possible contaminants in the environment and their detection limits, which cannot always cover the presence of low concentrations of pollutants. In addition, such approaches fail to produce a diagnostic insight concerning the type of stressor and at the same time they are unable to predict future impact early enough to avoid ecological damage. 

More advanced approaches propose the use of models and well-characterized multi-response systems to assess the responses of pollutants based on an understanding of the underlying mechanisms moving towards effect-based methods [3], taking into account complex mixture interactions [4] and explaining toxicity effects via adverse outcome pathways [5]. As a general approach, model species are exposed to single chemicals in laboratory studies to assess their individual mechanisms. However, since in the environment organisms are not just confronted with single pollutants but rather combinations of different stress factors in complex mixtures, their effects have been within the scope of current research, thereby identifying markers for pollution [6]. 

Focusing on the freshwater ecosystem, in this study, the impact of eight individual pollutants from diverse categories of commonly encountered pollutants were initially assessed individually on *Daphnia magna*. These chemicals represent four pharmaceuticals, two metals, one pesticide and a stimulant, which are commonly encountered threats for freshwater and marine species in the environment. Furthermore, for a realistic scenario, their composite mixture was assessed in non-lethal concentrations. Using lethality as a surrogate measure of toxicity and biochemical markers of physiology, individual toxicity and mixture effects for eight pollutants were assessed. To compare and validate how biochemical markers could provide meaningful information for environmental complex contamination, an in-depth assessment of the impact of the composite mixture at three stress intensities was subsequently performed on the metabolic level. 

## 2. Materials and Methods

### 2.1. Culturing of Daphnids and Toxicity Exposures

Daphnids were maintained in glass beakers in OECD media (final concentrations 0.29 g CaCl_2_.2H_2_O/L, 0.123 g MgSO_4_.7H_2_O/L, 0.065 g NaHCO_3_/L, 0.0058 g KCl/L, 2 μg Na_2_SeO_3_/L, pH 7.7) at a density of 80 adults per 4 L of media and under a 16h:8h of light:dark photoperiod at 20 °C [7]. For experiments, neonates (<24 h) were collected from the third brood of their mothers and cultured until four days old, and then used for experiments. Typically, in experiments with daphnids, acute toxicity is performed with neonates (<24 h); however, in several cases this has proven to be not reproducible, mainly because of the time window of neonate selection which extends to up to 24 h, thus resulting in a less homogenous population for experiments affecting toxicity [8]. Furthermore, as acute exposures are performed in the absence of food, the animals experience an additional stress of starvation which we avoided by allowing them to grow over a period of four days prior to exposure to the chemicals. Based on the selection of 24 h exposure periods, the chemicals were only added once following the general outlined procedure of the OECD guidelines [9]. The chemicals used in this study were aluminium (CAS 16828-11-8), lithium (CAS 7447-41-8), acetylsalicylic acid (CAS 50-78-2), diltiazem (CAS 33286-22-5), metformin (CAS 115-70-4), propranolol (CAS 318-98-9), glyphosate (CAS 1071-83-6) and nicotine (CAS 54-11-5). All chemicals were of highest purity >99.9%. 

**Figure 1:**
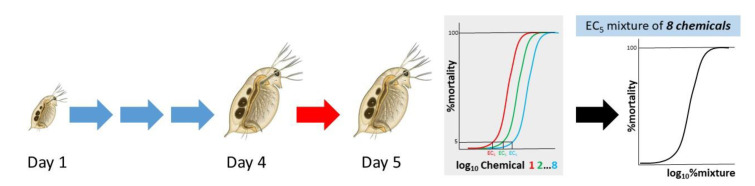
Experimental design. Four-day-old daphnids were exposed to eight chemicals individually for 24 h. Description of the combined mixture effect at EC_5_ was used to assess its toxicity at 24 h.

For exposures, 15 four-day-old animals were exposed to each chemical separately in a final volume of 100 mL OECD media with four replicates per concentration tested. Toxicity curves were plotted for 24 h exposures and EC values were calculated. A mixture was constructed for all chemicals and further assessed for its toxicity (Figure 1). All plots were calculated using the Four parameter logistic (4PL) model, following the equation Span = Top − Bottom and Y = Bottom + (Top-Bottom)/(1 + 10^((LogIC50-X)*HillSlope)), using the GraphPad software. The parameters top and bottom were commonly fixed to 100 and 0, accordingly.

### 2.2. Sample Homogenization and Biochemical Assays

Fifteen animals per biological replicate were pooled together and homogenized in 0.4 mL buffer using a pestle homogenizer. The homogenate was cleared by centrifugation (9000× *g* for 5 min at 5 °C), and the clear supernatant was collected and assessed immediately for enzyme activity. Phosphatases were assayed in 100 mM acetic acid pH 4.5 (for acid) or 100 mM boric acid pH 9.8 (for alkaline) using the substrate *p*-nitrophenyl phosphate and monitoring the production of *p*-nitrophenol at 405 nm after its alkalinization. Similarly, the activities of galactosidase and lipase were quantified by the generation of nitrophenol from the catalysis of *o*-nitrophenyl-β-galactoside or *p*-nitrophenyl butyrate, respectively, in phosphate buffer pH 7.2. Lactate dehydrogenase (LDH) activity was assessed from the consumption of NADH in a reaction with substrate of pyruvate (5 mM) at 340 nm [10]. Glutathione-S-transferase (GST) activity was measured by the formation of a complex between reduced glutathione with 1-chloro-2,4-dinitrobenzene in phosphate buffer pH 7.2 at 340 nm [11,12]. For reduced thiols, samples were homogenized in 100 mM acetic acid pH 4.5 and quantified following the protocol of Grintzalis et. al. [13]. Protein was quantified by a sensitive Bradford protocol [14].

### 2.3. Metabolomic Analysis

Fifteen animals were snap frozen in liquid nitrogen and analysed in the Metabolomics Core Technology Platform at the University of Heidelberg. For metabolite extraction, the frozen sample material was ground with a micropestle in 190 µL 100% methanol and incubated for 15 min at 70 °C with vigorous shaking. After the addition of 100 µL 100% chloroform, samples were shaken for 5 min at 37 °C. To separate polar and organic phases, 200 µL HPLC-grade water was added and samples were centrifuged for 10 min at 11,000× *g*. While avoiding the interphase containing cellular debris, 300 µL of the polar (upper) phase were transferred to a glass vial and dried using a vacuum concentrator (Eppendorf Concentrator Plus) without heating. Sequential on-line methoximation and silylation reactions were performed using an MPS autosampler (Gerstel, Mülheim Ruhr, Germany). Methoximation was performed by adding 20 µL 20 mg/mL methoxyamine hydrochloride (Sigma 226904, St. Louis, MO, USA) to pyridine (Sigma 270970) and incubation at 37 °C for 90 min in an MPS Agitator Unit (250 rpm). For silylation reactions, 45 µL of N-Methyl-N-(trimethylsilyl)trifluoroacetamide (MSTFA; Sigma 69479) was added and samples were incubated at 37 °C for 30 min with gentle shaking. Before injection, samples were incubated for 45 min at RT. For GC/MS analysis, a GC-ToF system was used consisting of an Agilent 7890 Gas Chromatograph (Agilent, Santa Clara) fitted with a Rxi-5Sil MS column (30 m × 0.25 mm × 0.25 µm; Restek) coupled to a Pegasus BT Mass Spectrometer (LECO). The GC was operated with an injection temperature of 250 °C and a 1 µL sample was injected with a split ratio of 10. The GC temperature program started with a 1 min hold at 40 °C followed by a 6 °C/min ramp up to 210 °C, a 20 °C/min ramp up to 330 °C and a bake-out at 330 °C for 5 min using Helium as a carrier gas with constant linear velocity. The ToF mass spectrometer was operated with ion source and interface temperatures of 250 °C, a solvent cut time of 9 min and a scan range (m/z) of 50–600 with an acquisition rate of 17 spectra/second. The ChromaTof v5.50 software (LECO Corporation, Saint Joseph, MI, USA) was used for data processing.

### 2.4. Statistical Analysis

The biochemical data were presented as mean ± standard deviation (SD) and were analysed and plotted with the GraphPad Prism software. For biochemical analysis, statistically significant differences were compared by Student’s *t*-test over unexposed control with a *p*-value of 0.05 for single chemical exposures with a null hypothesis that differences would be observed due to chance. For the different concentrations of the mixture, one-way ANOVA followed by comparisons with the control was performed and a test of a linear trend was validated. For metabolomic data (provided in Supplementary Materials), the values of peak area intensities were standardised by z scoring and then processed for multivariate statistical analysis with the freeware software Multi Experiment Viewer [15] to perform principal component analysis (PCA) and hierarchical clustering with Pearson distance metrics. A significant analysis of microarrays (SAM) between each exposed group and control was performed to identify significant fold changes in metabolites.

## 3. Results

### 3.1. Toxicity of Individual Chemicals and Their Mixture

**Figure 2:**
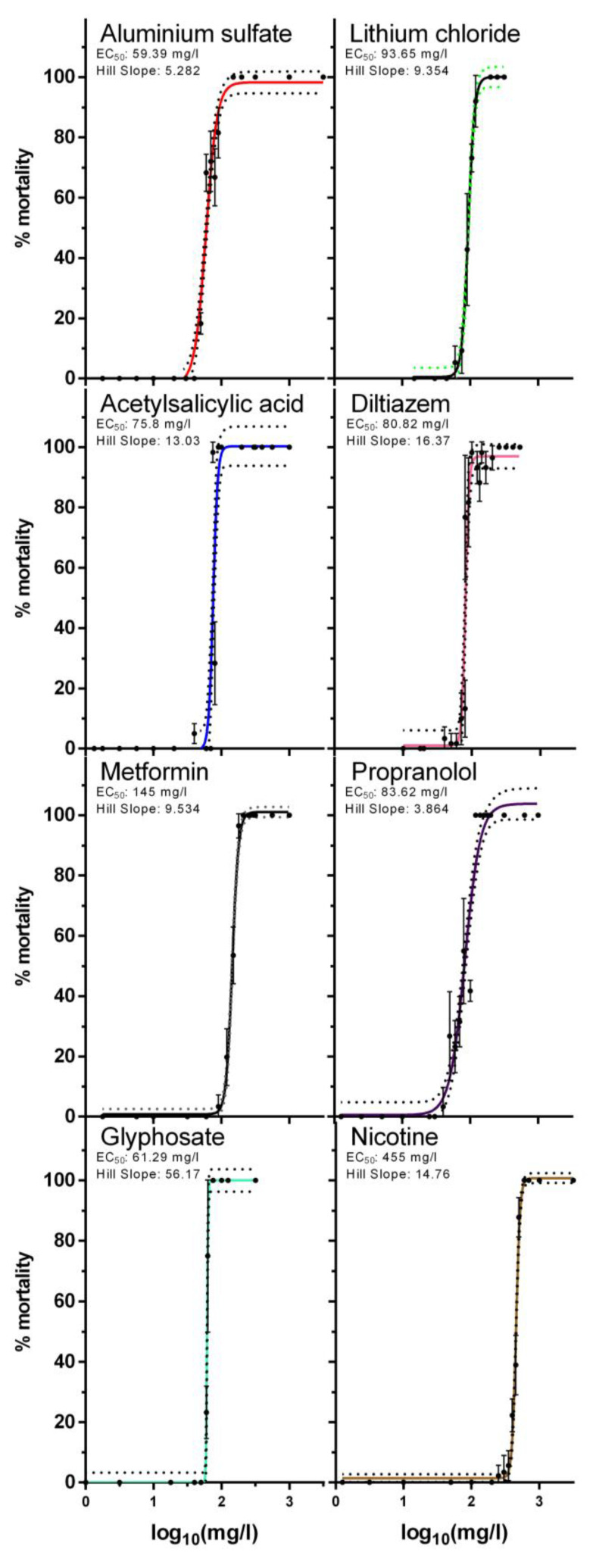
Acute toxicity curves for individual chemicals in this study. Data represent average ± standard deviation (N = 4 replicates).

**Figure 3:**
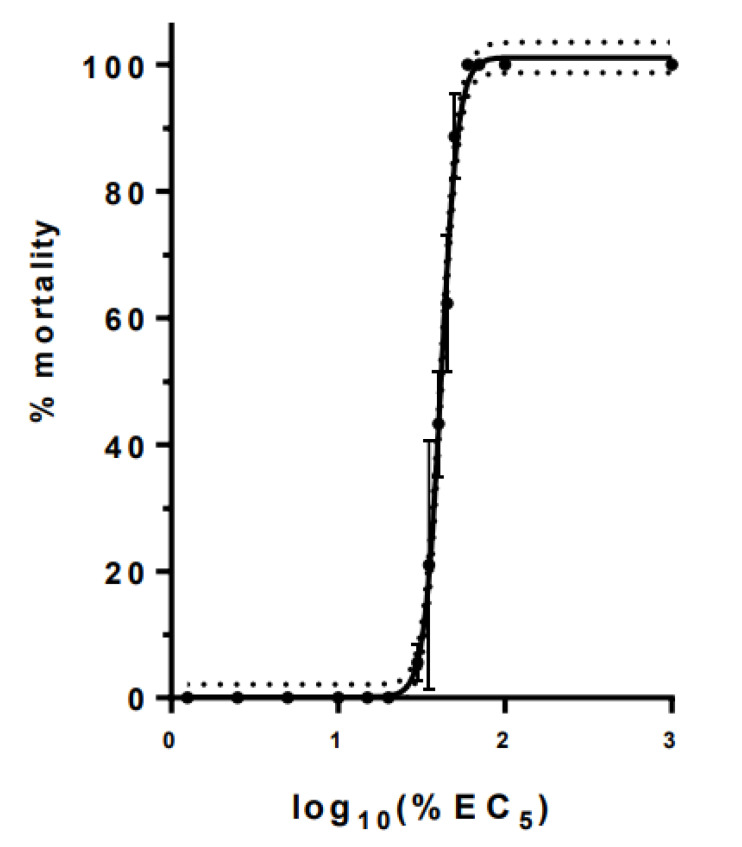
Acute toxicity curve of composite mixture of chemicals. Data represent average ± standard deviation (N = 6 replicates).

Acute exposure of daphnids to eight individual chemicals—Al, Li, acetylsalicylic acid, diltiazem, metformin, propranolol, glyphosate and nicotine—was assessed via toxicity curves (Figure 2) and the calculation of the effective concentration (EC) values (Table 1). As described, in this study we used older (four-day-old) daphnids and not the most sensitive neonates. The selection of this stage was made mainly because of the amount of tissue required but most importantly to avoid differences observed in some chemicals and their action because of the time window of the 0–24 h for collection of neonates. Specifically, it is well known that neonates have differences in toxicity responses as their collection could be from 0 to 24 h prior to the exposure, as shown for example for metals [8]. With this in mind and based on our previous experience, choosing a four-day stage as a starting point achieves better homogeneity allowing all individuals to grow to a level at which they will have a more unified response. Finally, in a freshwater population, all ages of daphnids are present, and therefore the neonate may serve as a more sensitive stage, but is not restrictive to the selection for this organism. As expected, the EC values recorded were in a similar order of magnitude but higher than reported EC_50_ values in the literature for neonates as neonates are more sensitive. In addition, a composite mixture in the ratio of the components’ EC_5_ (Table 1) was further explored for its toxicity in a range of dilutions to construct a full toxicity curve (Figure 3). For this toxicity curve the dose–response relationship was plotted as log_10_EC_5_, and low (10% EC_5_), medium (20% EC_5_), and high (30% EC_5_) concentrations were selected for the mixture exposures of daphnids for 24 h as non-lethal concentrations.

### 3.2. Enzyme Responses to Single Chemicals and Their Mixture

Exposure to individual stressors at EC_5_ revealed distinct responses in the activity of enzymes among the different pollutants (Table 2). Acetylsalicylic acid, followed by nicotine, induced fewer changes in enzyme activities, while on the other hand, metals were the most impactful stress by decreasing all enzyme activities with the exception of GST which was increased by aluminium and not lithium. Interestingly, there is not a specific pattern on the responses triggered as for example, propranolol only resulted in increases in activities of both phosphatases and peptidase. In relation to reduced thiols, four stressors (lithium, aluminium, nicotine and metformin) decreased their levels, while on the contrary, four chemicals (acetylsalicylic acid, propranolol, diltiazem and glyphosate) increased the levels of reduced thiols.

Exposure to the eight chemical mixtures resulted in dose-dependent changes for all enzyme biomarkers assessed (Table 3). Both acid (ACP) and alkaline (ALP) phosphatase activity was increased in the ranges of 18–37% and 36–41%, respectively, in a concentration-dependent manner relative to the intensity of the mixture. Furthermore, GST activity showed a trend to increase between 29% and 52%. On the other hand, β-galactosidase and lipase decreased between 12% and 42% dose-dependently with the stress intensity. Peptidase and lactate dehydrogenase, and reduced thiols, were also decreased in response to the concentration of the mixture, by 21% to 56%, respectively. The latter observed decrease in thiols could also be correlated with the increase in the activity of GST, which uses glutathione as a substrate to detoxify xenobiotics, and potentially other thiol-consuming enzymes.

### 3.3. The Metabolic Responses to Mixture Exposure

**Figure 4:**
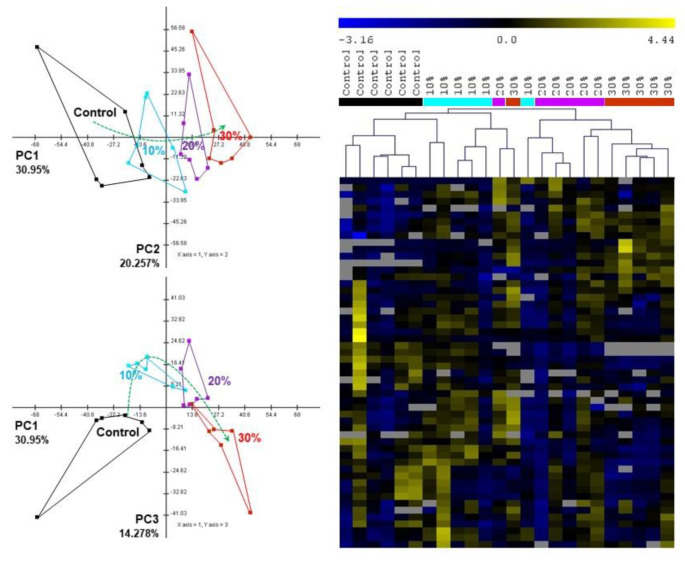
Multivariate statistical analysis of metabolomic data. PCA analysis and HCL shows the grouping and clustering of samples. The green arrow in PCA shows the gradual change in the metabolic profiles following the intensity of the mixture stress.

**Figure 5:**
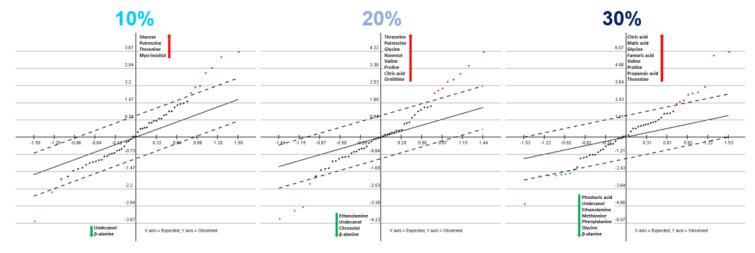
Significance Analysis of Microarrays (SAM) for each exposure compared with the unexposed control reveals the gradual change in the most significant metabolites.

An untargeted metabolomic analysis revealed a significant number of changes in the metabolism of daphnids upon exposure to the different intensities of the combined stress, thus supporting the observations in enzyme activities. Principal component analysis (PCA) and hierarchical clustering (HCL) show a clear grouping and clustering, respectively, of the metabolic profiles based on the intensity of the combined mixture stress (Figure 4). There is a clear trend (Figure 4 green arrow) of increase in intensity towards PC1. This is also supported by the significance analysis of microarrays analysis (SAM) (Figure 5), which allows the identification of significant changes based on the differential expression between sets of samples. Although this analysis is used in microarrays, it is also applicable to metabolomic data [16]. Statistically, this analysis provided a number of significantly up- or down-regulated metabolites with increasing stress intensities. For example, even from the low stress, undecanol and β-alanine were down-regulated, and with the increase in the intensity of the mixture stress to 20%, citronellol and ethanoloamine were also decreased, and at 30%, methionine, phenylalanine and glycine were added to the list of significantly decreased metabolites. On the other hand, all stress intensities up-regulated threonine and putrescine, while other metabolites of the TCA cycle (citric acid, fumaric acid), the urea cycle (ornithine) and amino acids (proline, valine) appear increased only at the middle (20%) and high (30%) intensity, thus indicating differences with the escalation of the stress effect.

## 4. Discussion

### 4.1. The Effects of Individual Stressors

There is a great number of chemicals simultaneously present in the environment, and a study of their effects separately is impossible in the actual environment. In most laboratory studies, the individual effects of single stressors are assessed in controlled experiments to understand their underlying mechanisms of toxicity.

Metal contamination is a major concern in aquatic ecosystems and, therefore, it is important to find reliable indicators of metal stress on aquatic organisms. In this study, aluminium and lithium were selected as two metal stressors commonly present in freshwater ecosystems. The adverse effects of both these metals have been studied in a variety of aquatic organisms such as sea urchins, fishes and snails [17], and in daphnids the reported EC_50_ for neonates is in a similar range to the EC_50_ reported in our study. In one study, aluminium exposure resulted in the differential expression of 155 genes [18] and its toxicity is believed to be mediated due to its capacity to strongly bind to phosphorus, thus reducing its availability. Ionic aluminium is able to inhibit extracellular phosphatases, and in our study this was the case for a decrease in the activity of acid phosphatase but not for alkaline phosphatase. Lithium has also been shown to exert toxic effects on aquatic organisms such as the fathead minnow and *Ceriodaphnia dubia*, implicating the role of other elements such as sodium decreasing lithium toxicity [17]. Furthermore, in relation to daphnids, lithium exposure resulted in 143 genes being differentially expressed, some even by over three-fold [19]. In some studies, lithium led to significant metabolite variations, specifically in amino acids as well as uracil and the osmolyte glycerophosphocholine, thus revealing toxicity-mediated effects by impairing energy production and ionoregulation [20]. In the present study, both metals had a significant impact through decreasing most biochemical markers when applied independently. Furthermore, the observed increase in GST activity upon aluminium exposure has also been reported by others as a response to heavy metals in plants [21] and to toxins in daphnids [22].

With the demographic trend of an ageing of populations and the high accessibility of medication and drugs, pharmaceuticals have been highlighted as a class of emerging pollutants. This in turn is also amplified by their improper disposal and limited removal from waste water treatment plants. Non-steroidal anti-inflammatory drugs (NSAIDs) are probably the most widely used medication worldwide. In our study, acetylsalicylic acid, best known as aspirin, was studied for its impact on daphnids as a representative NSAID. Acetylsalicylic acid has been reported to decrease survival rates, fecundity and growth in daphnids [23], and the reported EC_50_ values are similar to the ones observed in this study. In daphnids, it has been shown that acetylsalicylic acid mediates its toxicity via an induction of oxidative stress with an increase in lipid and protein oxidation which is accompanied by DNA damage. In response to the aforementioned effects on oxidative stress, changes in antioxidant enzyme activities such as superoxide dismutase and catalase have been recorded [24]. A more holistic approach revealed that three genes are significantly up-regulated and four genes are significantly downregulated in response to acetylsalicylic acid [25]. However, in our study, acetylsalicylic acid had a less significant impact, decreasing only the activity of peptidase.

Diltiazem is a non-dihydropyridine calcium channel blocker prescribed to treat high blood pressure and to control angina [26]. Diltiazem inhibits calcium influx into both cardiac and smooth muscles during depolarisation, and as such, it is most probable that toxic effects would occur through the disruption of regulation of cellular calcium levels. Maintenance of appropriate calcium levels is important for many physiological processes in all organisms, including daphnids. At low concentrations of 500 ng/L, diltiazem has been shown to increase the heart rate of *Daphnia magna*, along with oxygen consumption, thus resulting in energy imbalance and a higher demand for energy [27]. Although in our study we did not assess phenotypic endpoints, diltiazem decreased GST activity and increased alkaline phosphatase and lipase. The latter observed increase in lipase activity could be seen as consistent with the decrease in lipids reported in other studies [27].

Metformin is a medication used under many brand names and is the most common drug prescribed to treat type 2 diabetes and polycystic ovary syndrome. Because of its wide audience of patients, it is very much consumed and, thus, commonly present in the aquatic ecosystem [28]. Metformin may interact with a variety of molecular targets across species [29]. In relation to its actions on aquatic species, there is literature on its impact on fecundity and behaviour in fish [30,31] and therefore, metformin has been attributed as an endocrine disruptor [32]. There are few biochemical data available for daphnids; however, the mechanism of action of metformin has been linked to the induction of the hypoxia-inducible factor (HIF) α and β genes [33]. In our study, metformin proved to be a strong stressor and decreased all enzyme activities simultaneously, thus showing a significant impact on daphnids.

Propranolol is a drug that belongs to the category of β-blockers, which are prescribed for the treatment of stable coronary ischaemic disease [34]. Propranolol has been detected in the freshwater environment in significant high levels, and although it is designed for human therapeutic usage, it exerts its effects on non-target organisms. As a drug with a significant bioaccumulation effect, propranolol at a very low concentration has been shown to bioaccumulate in daphnids at up to 1.6× its original concentration over 10 generations [35]. Propranolol is toxic to neonates with an ambient EC_50_ of 7.5 mg/l [36], which is significantly lower than the one determined in our study, which could be attributed to the higher sensitivity of neonates when compared with the four-day-old daphnids used here. Propranolol may exert its toxic effects in daphnids in an organ-specific manner, such as reducing the heart rate [37], in addition to decreasing fecundity (at 0.22 to 0.44 mg/L) and completely inhibiting it (at 0.88 mg/L). It is worth noting that in transgenerational exposures, the second generation of daphnids was less sensitive to propranolol [38] although in other studies even subtle environmentally relevant concentrations may induce physiological changes [39]. Propranolol has been reported to increase GST activity and inhibit glutathione peroxidase (GPx), an enzyme that removes peroxyl radicals and hydroperoxides [40], thus affecting the antioxidant defence system of daphnids. Interestingly, in our study, propranolol increased the activity of both phosphatases and peptidase.

N-phosphonomethyl glycine is a well-known herbicide called glyphosate, which has been employed extensively in agriculture, and thus can be found in the environment as a consequence of agricultural or urban run-off and leaching into local surface waters [41]. There have long been concerns over its implications in the environment and for public health. Glyphosate has been shown to cause morphological alterations in zooplanctonic organisms and crustaceans [42], and to impact carbon and fat metabolism and the microbiome [43] as well as the heart rate of daphnids [44]. Our results support these findings, as an increase in lipase and peptidase was observed along with a decrease in alkaline phosphatase and GST. The latter is in agreement with similar decreases in fish and could be explained as failure of detoxification processes and development of oxidative stress [45]. In addition, recent studies on daphnids showed that glyphosate exposure can modify the mRNA transcription and enzymatic activity of GST and lipid peroxidation [46] and even exacerbate its toxicity in the presence of microplastics [47].

The last chemical studied was a stimulant, nicotine, which is widely consumed within a number of products in daily life and is a key ingredient of tobacco. There is a general lack of data on the impact of nicotine on freshwater organisms, and many existing studies focus solely on mortality or heart-rate as physiology endpoints. Nicotine has been found to reduce fecundity in daphnids by decreasing the number of neonates released per individual. It also triggers the production of male offspring [48] and induces antenna, carapace and spine malformations [49] in daphnids. In our study, nicotine decreased the activities of peptidase, LDH and GST, and the latter could indicate a possible induction of stress in daphnids.

### 4.2. Mixture Effects and Omics in Toxicology

Organisms in their natural environments are exposed to composite mixtures of several individual chemicals at low concentrations. This is an issue, as chemical interactions within these mixtures can result in unintuitive results. The individual components were mixed at an EC_5_ ratio; however, the mixture, as expected, was significantly more toxic than its constituents alone. This is known as synergy, meaning that the components act together, and this poses a major problem in the context of environmental risk assessments. These results are not easily predicted, so new methods are needed to understand the mechanisms and interactions within these mixtures. Effect-based methods gain more attention in the literature as complementary strategies to the chemical analytical characterisation of complex pollution patterns and they can provide new metrics for pollution assessment [50].

For mixture prediction, there exist two concepts—Concentration Addition (CA) and Independent Action (IA)—that allow the calculation of expectable combined effects based on individual components’ bioactivities and mixture exposure knowledge. Both concepts are based on knowledge of the single-compound toxicities and the assumption of no interaction. CA assumes that the individual components behave as simple dilutions of one another, which is commonly interpreted as being the case for compounds of a mixture sharing a strictly similar mechanism of action. However, IA supports the completely independent action of chemicals, which is commonly interpreted as the compounds of a mixture having dissimilar mechanisms of action. In a recent study on daphnids where mortality was used as the only endpoint, four contaminants (sodium fluoride, boric acid, ammonium hydroxide and acetaminophen) were assessed in mixtures. Regardless of the assumption of dose- or response-additivity, independent action slightly outperformed concentration addition in most of the combinations of these multiple-class compounds [51]. In this study, we considered that individual responses were low but we did not perform any analysis of non-interactive mixture modelling, but rather we chose to elaborate for observation of biochemical markers of enzyme activities, in order to understand the biological responses of daphnids when exposed to low (10% EC5), medium (20% EC5) and high (30% EC5) concentrations of the composite chemical mixture. As it was hypothesised, a clear dose response in connection to the stress intensity was observed, which could be attributed to a synergy effect in the above meaning. However, a more in-depth level of knowledge would require more sophisticated measurements. Holistic or omics methodologies are widely used in toxicological research and have a pivotal role in the understanding of mechanisms of toxicity [52]. These analytical approaches extend from the epigenome to the transcriptome, proteome and metabolome level. Metabolomics is the study of low-molecular-weight metabolites and metabolic pathways within living organisms, and is a fast-evolving research field with pioneering investigations in relation to the biochemical responses to toxicants. Metabolism is a decisive parameter for physiology and is of central importance for adaptation of all life forms. Therefore, the unique strength of metabolomics is that it measures the functional status of an organism as the alterations in metabolic levels are the primary adaptation mechanism in organisms [53]. This is because by nature metabolism responds fast to environmental stimuli which allows the analysis and interpretation of the organism’s response at a molecular pathway level. In our experiments, alterations in the TCA cycle, the urea cycle and metabolism of amino acids were highlighted as perturbed with intensity of stress. Although our analysis was a preliminary discovery analysis and not targeted to any pathway, targeted methods have identified perturbations in specific pathways [54] or mapped these changes on specific tissues [55] in these crustaceans. Furthermore, this study focused on a composite mixture as a stress pool and not on a specific chemical; however, lithium, for example, has been recently assessed in its nanoparticle form to affect amino acid, starch and glucose metabolism, which could also be reflected in our study from changes in the relevant catabolic enzymes [56].

Metabolomic analysis in daphnids has been highlighted as a key mechanistic tool for environmental monitoring [57] and can provide valuable fitness information at the molecular level [58]. Our study verified that simple biochemical markers of enzyme activities show similar patterns in changes with sensitive holistic metabolomic analysis. In this context, simple enzyme activity endpoints can be used as a first step to evaluate and design metabolomic detailed studies safely. To our knowledge, this is the first study where a direct link was observed in daphnids between the activities of key enzymes relevant to the physiology of daphnids and metabolomic analysis, thus verifying the employment of the first as potent markers in toxicology assessment.

## Figures and Tables

**Table 1:** EC values (in mg/L) from toxicity curves and mixture ratio at EC_5_ *.

Chemical	EC_50_	Hill Slope	EC_5_	% in Mixture
Aluminium sulfate hexadecahydrate	59.4	5.282	34	4.53
Lithium chloride	93.7	9.354	68.4	9.11
Acetylsalicylic acid	75.8	13.03	60.5	8.06
Diltiazem hydrochloride	80.8	16.37	67.5	8.99
Metformin	145	9.534	106.5	14.19
Propranolol	83.6	3.864	39	5.19
Glyphosate	61.3	56.17	1.69	0.225
Nicotine	455	14.76	373	49.69

* presented precision does not signal significance but serves the purpose for reusability.

**Table 2:** Biochemical markers of daphnid physiology upon exposure to a mixture of eight chemicals. Data represent mean ± standard deviation (N = 4) of enzyme activity. Enzyme activity was expressed as units/mg protein for lactate dehydrogenase (LDH), lipase, β-galactosidase and phosphatases, as munits/mg protein for GST, and for reduced thiols in nmoles/mg protein. Bold font indicates statistically significant difference by Student’s *t*-test compared with the unexposed control.

	Control	Li	Al	Acetyl Salicylic Acid	Propranolol	Diltiazem	Glyphosate	Nicotine	Metformin
ALP	7.53 ± 0.88	**2.2 ± 0.21** **(−71%)**	8.8 ± 0.31	8.13 ± 0.68	**13.98 ± 1.19** **(+84%)**	**10.6 ± 0.34** **(+41%)**	**5.42 ± 0.53** **(−28%)**	7.77 ± 0.79	**4.7 ± 1.18** **(−38%)**
ACP	5 ± 0.69	**1.8 ± 0.07** **(−64%)**	**3.4 ± 0.13** **(−32%)**	5.73 ± 0.09	**7.44 ± 0.46** **(+49%)**	5.7 ± 0.24	5 ± 0.21	4.38 ± 0.3	**3.17 ± 0.71** **(−37%)**
βGAL	11.63 ± 0.2	**1.85 ± 0.1** **(−84%)**	**3.13 ± 0.05** **(−73%)**	12.36 ± 0.87	9.96 ± 1.14	11.7 ± 1.02	11.7 ± 0.42	11.1 ± 1.25	**4.72 ± 0.71** **(−59%)**
Lipase	165 ± 10.1	**50.2 ± 2.3** **(−70%)**	**104.6 ± 13.5** **(−37%)**	181.64 ± 3.7	153 ± 16.4	**190 ± 12.5 (+15%)**	**187.8 ± 9.6** **(+14%)**	169.4 ± 11.8	**84.2 ± 16.7** **(−49%)**
Peptidase	286 ± 21.8	**53 ± 10.7** **(−82%)**	**158 ± 11.8** **(−45%)**	**240 ± 17.6** **(−16%)**	**387 ± 41.5 (+35%)**	291 ± 41.8	**340 ± 8.9** **(+19%)**	**217 ± 27.5** **(−24%)**	**138 ± 27.8** **(−52%)**
LDH	80.32 ± 6.52	**63.8 ± 5.33** **(−21%)**	83 ± 5.22	84.6 ± 4.33	77.7 ± 2.51	86.42 ± 7.88	72.59 ± 6.85	**65.5 ± 3.15** **(−18%)**	**67.4 ± 4.91** **(−16%)**
GST	212 ± 21.7	**34.3 ± 19.7** **(−84%)**	**274 ± 7.6** **(+29%)**	198.6 ± 6.6	215.6 ± 24.5	**151.4 ± 6** **(−29%)**	**134.7 ± 7.2** **(−37%)**	**155.6 ± 4.8** **(−27%)**	254.8 ± 9.2
Reduced thiols	64.9 ± 3.5	**36.7 ± 1.2** **(−43%)**	**51.9 ± 1.7** **(−20%)**	**79.9 ± 2** **(+23%)**	**74.1 ± 2.5** **(+14%)**	**70.5 ± 2.2** **(+8.6%)**	**73.6 ± 3.9** **(+13.5%)**	**50.7 ± 1.3** **(−22%)**	**57.7 ± 3.2** **(−11%)**

**Table 3:** Biochemical markers of daphnid physiology upon exposure to a mixture of eight chemicals. Data represent mean ± standard deviation (N = 4) of enzyme activity. Enzyme activity was expressed as units/mg protein for lactate dehydrogenase (LDH), lipase, β-galactosidase and phosphatases, as munits/mg protein for GST, and for reduced thiols in nmoles/mg protein. Bold font indicates statistically significant difference by one-way ANOVA followed by comparison with the unexposed control.

	Control	10%	20%	30%
ALP	8.28 ± 0.19	**11.67 ± 1.01 (+41%)**	**11.64 ± 0.32 (+41%)**	**11.22 ± 1.19 (+36%)**
ACP	3.08 ± 0.14	**3.62 ± 0.44 (+18%)**	**4.05 ± 0.27 (+31%)**	**4.29 ± 0.45 (+37%)**
βGAL	3.62 ± 0.06	**3.19 ± 0.09 (−12%)**	**2.6 ± 0.13 (−28%)**	**2.11 ± 0.09 (−42%)**
Lipase	17.73 ± 0.59	**15.34 ± 1.31 (−13%)**	**10.43 ± 1.18 (−41%)**	**10.85 ± 1.24 (−39%)**
Peptidase	95.65 ± 4.44	93.09 ± 12.29	**73.8 ± 6.81 (−23%)**	**77.18 ± 6.58 (−20%)**
LDH	54.79 ± 2.32	50.44 ± 7.07	**32.9 ± 10.11 (−40%)**	**18.41 ± 4.3 (−67%)**
GST	149.52 ± 3.36	169.14 ± 13.02	**193.41 ± 7.56 (+29%)**	**227.61 ± 23.68 (+52%)**
Reduced thiols	201.44 ± 31.76	**158.82 ± 12.12 (−21%)**	**115.69 ± 17.18 (−43%)**	**88.69 ± 35.97 (−56%)**

## Data Availability

Not applicable.

## Supplementary Materials

The following supporting information can be downloaded at: https://www.mdpi.com/article/10.3390/toxics10100604/s1, Table S1: Metabolomic data.

## References

[1] Ahmed U., Mumtaz R., Anwar H., Mumtaz S., Qamar A.M. (2019). Water quality monitoring: From conventional to emerging technologies. Water Supply.

[2] Mardrid Y., Zayas Z.P. (2007). Water sampling: Traditional methods and new approaches in water sampling strategy. Trends Anal. Chem..

[3] Brack W., Aissa S.A., Backhaus T., Dulio V., Escher B.I., Faust M., Hilscherova K., Hollender J., Hollert H., Müller C. (2019). Effect-based methods are key. The European Collaborative Project SOLUTIONS recommends integrating effect-based methods for diagnosis and monitoring of water quality. Environ. Sci. Eur..

[4] Altenburger R., Brack W., Burgess R.M., Busch W., Escher B.I., Focks A., Mark Hewitt L., Jacobsen B.N., de Alda M.L., Ait-Aissa S. (2019). Future water quality monitoring: Improving the balance between exposure and toxicity assessments of real-world pollutant mixtures. Environ. Sci. Eur..

[5] Groh K.J., Carvalho R.N., Chipman J.K., Denslow N.D., Halder M., Murphy C.A., Roelofs D., Rolaki A., Schirmer K., Watanabe K.H. (2015). Development and application of the adverse outcome pathway framework for understanding and predicting chronic toxicity: I. Challenges and research needs in ecotoxicology. Chemosphere.

[6] Escher B.I., Stapleton H.M., Schymanski E.L. (2020). Tracking complex mixtures of chemicals in our changing environment. Science.

[7] Grintzalis K., Dai W., Panagiotidis K., Belavgeni A., Viant M.R. (2017). Miniaturising acute toxicity and feeding rate measurements in Daphnia magna. Ecotoxicol. Environ. Saf..

[8] Traudt E.M., Ranville J.F., Meyer J.S. (2017). Effect of age on acute toxicity of cadmium, copper, nickel, and zinc in individual-metal exposures to Daphnia magna neonates. Environ. Toxicol. Chem..

[9] OECD (2004). Test No. 202: Daphnia sp. Acute Immobilisation Test.

[10] Worthington K., Worthington V. Worthington Enzyme Manual. https://www.worthington-biochem.com/index/manual.html.

[11] Tang S.S., Lin C.C., Chang G.G. (1996). Metal-catalyzed oxidation and cleavage of octopus glutathione transferase by the Cu(II)-ascorbate system. Free Radic. Biol. Med..

[12] Warholm M., Guthenberg C., Mannervik B., Pacifici G.M., Rane A. (1981). Glutathione S-transferases in human fetal liver. Acta Chem. Scandinavica. Ser. B: Org. Chem. Biochem..

[13] Grintzalis K., Papapostolou I., Zisimopoulos D., Stamatiou I., Georgiou C.D. (2014). Multiparametric protocol for the determination of thiol redox state in living matter. Free Radic. Biol. Med..

[14] Grintzalis K., Georgiou C.D., Schneider Y.J. (2015). An accurate and sensitive Coomassie Brilliant Blue G-250-based assay for protein determination. Anal. Biochem..

[15] Saeed A.I., Sharov V., White J., Li J., Liang W., Bhagabati N., Braisted J., Klapa M., Currier T., Thiagarajan M. (2003). TM4: A free, open-source system for microarray data management and analysis. BioTechniques.

[16] Diercks D.B., Owen K.P., Kline J.A., Sutter M.E. (2016). Urine metabolomic analysis to detect metabolites associated with the development of contrast induced nephropathy. Clin. Exp. Emerg. Med..

[17] Kszos L.A., Beauchamp J.J., Stewart A.J. (2003). Toxicity of Lithium to Three Freshwater Organisms and the Antagonistic Effect of Sodium. Ecotoxicology.

[18] Brun N.R., Fields P.D., Horsfield S., Mirbahai L., Ebert D., Colbourne J.K., Fent K. (2019). Mixtures of Aluminum and Indium Induce More than Additive Phenotypic and Toxicogenomic Responses in Daphnia magna. Environ. Sci. Technol..

[19] Kim H.J., Yang J.H., Kim H.S., Kin Y.J., Seo Y.R. (2017). Exploring potential biomarker responses to lithium in *Daphnia magna* from the perspectives of function and signaling networks. Mol. Cell. Toxicol..

[20] Nagato E.G., D’Eon J.C., Lankadurai B.P., Poirier D.G., Reiner E.J., Simpson A.J., Simpson M.J. (2013). (1)H NMR-based metabolomics investigation of Daphnia magna responses to sub-lethal exposure to arsenic, copper and lithium. Chemosphere.

[21] Kumar S., Trivedi P.K. (2018). Glutathione S-Transferases: Role in Combating Abiotic Stresses Including Arsenic Detoxification in Plants. Front. Plant Sci..

[22] Lyu K., Gu L., Li B., Lu Y., Wu C., Guan H., Yang Z. (2016). Stress-responsive expression of a glutathione S-transferase (delta) gene in waterflea Daphnia magna challenged by microcystin-producing and microcystin-free Microcystis aeruginosa. Harmful Algae.

[23] Marques C.R., Abrantes N., Goncalves F. (2004). Life-history traits of standard and autochthonous cladocerans: II. Acute and chronic effects of acetylsalicylic acid metabolites. Environ. Toxicol..

[24] Gomez-Olivan L.M., Galar-Martinez M., Islas-Flores H., Garcia-Medina S., SanJuan-Reyes N. (2014). DNA damage and oxidative stress induced by acetylsalicylic acid in Daphnia magna. Comp. Biochem. Physiol. Toxicol. Pharmacol. CBP.

[25] Bang S.H., Hong N.H., Ahn J.Y., Sekhon S.S., Kim Y.-H., Min J. (2015). Proteomic analysis of *Daphnia magna* exposed to caffeine, ibuprofen, aspirin and tetracycline. Toxicol. Environ. Health Sci..

[26] Crawford M.H. (1985). Effectiveness of diltiazem for chronic stable angina pectoris. Acta Pharmacol. Et Toxicol..

[27] Steinkey D., Lari E., Woodman S.G., Steinkey R., Luong K.H., Wong C.S., Pyle G.G. (2019). The effects of diltiazem on growth, reproduction, energy reserves, and calcium-dependent physiology in Daphnia magna. Chemosphere.

[28] Oosterhuis M., Sacher F., Ter Laak T.L. (2013). Prediction of concentration levels of metformin and other high consumption pharmaceuticals in wastewater and regional surface water based on sales data. Sci. Total Environ..

[29] Ambrosio-Albuquerque E.P., Cusioli L.F., Bergamasco R., Sinopolis Gigliolli A.A., Lupepsa L., Paupitz B.R., Barbieri P.A., Borin-Carvalho L.A., de Brito Portela-Castro A.L. (2021). Metformin environmental exposure: A systematic review. Environ. Toxicol. Pharmacol..

[30] Alla L.N.R., Monshi M., Siddiqua Z., Shields J., Alame K., Wahls A., Akemann C., Meyer D., Crofts E.J., Saad F. (2021). Detection of endocrine disrupting chemicals in Danio rerio and Daphnia pulex: Step-one, behavioral screen. Chemosphere.

[31] Niemuth N.J., Jordan R., Crago J., Blanksma C., Johnson R., Klaper R.D. (2015). Metformin exposure at environmentally relevant concentrations causes potential endocrine disruption in adult male fish. Environ. Toxicol. Chem..

[32] Elizalde-Velazquez G.A., Gomez-Olivan L.M. (2020). Occurrence, toxic effects and removal of metformin in the aquatic environments in the world: Recent trends and perspectives. Sci. Total Environ..

[33] Sheng B., Liu J., Li G.H. (2012). Metformin preconditioning protects Daphnia pulex from lethal hypoxic insult involving AMPK, HIF and mTOR signaling. Comp. Biochem. Physiol. Part B Biochem. Mol. Biol..

[34] Kloner R.A., Fishbein M.C., Cotran R.S., Braunwald E., Maroko P.R. (1977). The effect of propranolol on microvascular injury in acute myocardial ischemia. Circulation.

[35] Jeong T.Y., Kim T.H., Kim S.D. (2016). Bioaccumulation and biotransformation of the beta-blocker propranolol in multigenerational exposure to Daphnia magna. Environ. Pollut..

[36] Cleuvers M. (2003). Aquatic ecotoxicity of pharmaceuticals including the assessment of combination effects. Toxicol. Lett..

[37] Jeong T.Y., Yoon D., Kim S., Kim H.Y., Kim S.D. (2018). Mode of action characterization for adverse effect of propranolol in Daphnia magna based on behavior and physiology monitoring and metabolite profiling. Environ. Pollut..

[38] Dzialowski E.M., Turner P.K., Brooks B.W. (2006). Physiological and reproductive effects of beta adrenergic receptor antagonists in Daphnia magna. Arch. Environ. Contam. Toxicol..

[39] Jeong T.Y., Kim H.Y., Kim S.D. (2015). Multi-generational effects of propranolol on Daphnia magna at different environmental concentrations. Environ. Pollut..

[40] Oliveira L.L., Antunes S.C., Goncalves F., Rocha O., Nunes B. (2015). Evaluation of ecotoxicological effects of drugs on Daphnia magna using different enzymatic biomarkers. Ecotoxicol. Environ. Saf..

[41] Noori J.S., Dimaki M., Mortensen J., Svendsen W.E. (2018). Detection of Glyphosate in Drinking Water: A Fast and Direct Detection Method without Sample Pretreatment. Sensors.

[42] Gustinasari K., Slugocki L., Czerniawski R., Pandebesie E.S., Hermana J. (2021). Acute toxicity and morphology alterations of glyphosate-based herbicides to Daphnia magna and Cyclops vicinus. Toxicol. Res..

[43] Suppa A., Kvist J., Li X., Dhandapani V., Almulla H., Tian A.Y., Kissane S., Zhou J., Perotti A., Mangelson H. (2020). Roundup causes embryonic development failure and alters metabolic pathways and gut microbiota functionality in non-target species. Microbiome.

[44] Duan K.R., Kish A., Kish L., Faletra P., Salmon K.M. (2019). The Impact of Glyphosate-Based Herbicides and Their Components on Daphnia Magna. bioRxiv.

[45] Palas S., Sandipan P., Kumar A.k., Apurba Ratan G. (2014). Biochemical effects of glyphosate based herbicide, Excel Mera 71 on enzyme activities of acetylcholinesterase (AChE), lipid peroxidation (LPO), catalase (CAT), glutathione-S-transferase (GST) and protein content on teleostean fishes. Ecotoxicol. Environ. Saf..

[46] Zhang H.C., Yang Y.J., Ma K.X., Shi C.Y., Chen G.W., Liu D.Z. (2020). A novel sigma class glutathione S-transferase gene in freshwater planarian Dugesia japonica: Cloning, characterization and protective effects in herbicide glyphosate stress. Ecotoxicology.

[47] Zocchi M., Sommaruga R. (2019). Microplastics modify the toxicity of glyphosate on Daphnia magna. Sci. Total Environ..

[48] Oropesa A.L., Floro A.M., Palma P. (2017). Toxic potential of the emerging contaminant nicotine to the aquatic ecosystem. Environ. Sci. Pollut. Res. Int..

[49] Chen K.-F., Huang S.-Y., Chung Y.-T., Wang K.-S., Wang C.-K., Chang S.-H. (2018). Detoxification of nicotine solution using Fe0-based processes: Toxicity evaluation by *Daphnia magna* neonate and embryo assays. Chem. Eng. J..

[50] Altenburger R., Scholze M., Busch W., Escher B.I., Jakobs G., Krauss M., Krüger J., Neale P.A., Ait-Aissa S., Almeida A.C. (2018). Mixture effects in samples of multiple contaminants—An inter-laboratory study with manifold bioassays. Environ. Int..

[51] Silva A.R.R., Gonçalves S.F., Pavlaki M.D., Morgado R.G., Soares A., Loureiro S. (2022). Mixture toxicity prediction of substances from different origin sources in Daphnia magna. Chemosphere.

[52] Harrill J.A., Viant M.R., Yauk C.L., Sachana M., Gant T.W., Auerbach S.S., Beger R.D., Bouhifd M., O’Brien J., Burgoon L. (2021). Progress towards an OECD reporting framework for transcriptomics and metabolomics in regulatory toxicology. Regul. Toxicol. Pharmacol. RTP.

[53] Roessner U., Bowne J. (2009). What is metabolomics all about?. BioTechniques.

[54] Labine L.M., Simpson M.J. (2021). Targeted Metabolomic Assessment of the Sub-Lethal Toxicity of Halogenated Acetic Acids (HAAs) to Daphnia magna. Metabolites.

[55] Smith M.J., Weber R.J.M., Viant M.R. (2022). Spatially Mapping the Baseline and Bisphenol-A Exposed Daphnia magna Lipidome Using Desorption Electrospray Ionization-Mass Spectrometry. Metabolites.

[56] Niemuth N.J., Curtis B.J., Laudadio E.D., Sostare E., Bennett E.A., Neureuther N.J., Mohaimani A.A., Schmoldt A., Ostovich E.D., Viant M.R. (2021). Energy Starvation in Daphnia magna from Exposure to a Lithium Cobalt Oxide Nanomaterial. Chem. Res. Toxicol..

[57] Jeong T.-Y., Simpson M.J. (2019). Daphnia magna metabolic profiling as a promising water quality parameter for the biological early warning system. Water Res..

[58] Taylor N.S., Gavin A., Viant M.R. (2018). Metabolomics Discovers Early-Response Metabolic Biomarkers that Can Predict Chronic Reproductive Fitness in Individual Daphnia magna. Metabolites.

